# Cue-dependent effects of VR experience on motion-in-depth sensitivity

**DOI:** 10.1371/journal.pone.0229929

**Published:** 2020-03-09

**Authors:** Jacqueline M. Fulvio, Mohan Ji, Lowell Thompson, Ari Rosenberg, Bas Rokers

**Affiliations:** 1 Department of Psychology, University of Wisconsin–Madison, Madison, Wisconsin, United States of America; 2 Department of Neuroscience, School of Medicine and Public Health, University of Wisconsin–Madison, Madison, Wisconsin, United States of America; Johns Hopkins University, UNITED STATES

## Abstract

The visual system exploits multiple signals, including monocular and binocular cues, to determine the motion of objects through depth. In the laboratory, sensitivity to different three-dimensional (3D) motion cues varies across observers and is often weak for binocular cues. However, laboratory assessments may reflect factors beyond inherent perceptual sensitivity. For example, the appearance of weak binocular sensitivity may relate to extensive prior experience with two-dimensional (2D) displays in which binocular cues are not informative. Here we evaluated the impact of experience on motion-in-depth (MID) sensitivity in a virtual reality (VR) environment. We tested a large cohort of observers who reported having no prior VR experience and found that binocular cue sensitivity was substantially weaker than monocular cue sensitivity. As expected, sensitivity was greater when monocular and binocular cues were presented together than in isolation. Surprisingly, the addition of motion parallax signals appeared to cause observers to rely almost exclusively on monocular cues. As observers gained experience in the VR task, sensitivity to monocular and binocular cues increased. Notably, most observers were unable to distinguish the direction of MID based on binocular cues above chance level when tested early in the experiment, whereas most showed statistically significant sensitivity to binocular cues when tested late in the experiment. This result suggests that observers may discount binocular cues when they are first encountered in a VR environment. Laboratory assessments may thus underestimate the sensitivity of inexperienced observers to MID, especially for binocular cues.

## Introduction

Three-dimensional (3D) visual perception is made more precise by integrating multiple cues (e.g., [1–8]). In the case of discriminating 3D motion, observers rely on monocular cues such as optic flow, as well as object size and density changes [9–11]. They can also rely on binocular cues including changing disparity and interocular velocity differences [12–17]. However, sensitivity to different motion-in-depth (MID) cues varies across observers [16, 18–23]. Even within observers, sensitivity to MID cues can vary considerably across the visual field [23–26]. In addition, sensitivity to binocular MID cues is often relatively poor in the laboratory, especially for observers with little prior psychophysical experience [23, 27,28]. Applied research further suggests that displays which present binocular cues, such as 3D TVs, have little effect on the perceptual experience [29]. These findings call the relative importance of binocular cues to 3D motion perception into question and have substantial implications for the 3D media industry.

The appearance of poor sensitivity to binocular motion cues may be related to prior experience. A typical observer has extensive experience with visual displays (e.g., televisions, tablets, and cell phones) which convey 3D motion through monocular but not binocular cues. The presentation of MID in 3D displays also requires the apparent movement of visual elements through a physical screen, which conflicts with the real world experience that solid objects do not seamlessly pass through each other. As such, when observers are first presented with binocular MID cues in a display, the cues may simply be discounted. Thus, poor sensitivity to binocular cues may not be an inherent feature of visual processing, but instead reflect cue discounting.

We recently found that the performance of inexperienced observers on a virtual reality (VR)-based MID task only improved with feedback [30]. There, the stimuli always contained monocular and binocular cues, and in a separate condition, also contained motion parallax cues. Thus, the effect of feedback on sensitivity to individual MID cues presented in a VR display could not be determined. Here we evaluated how the sensitivity of observers to individual MID cues depended on feedback-based VR experience.

We presented MID stimuli in a head-mounted VR display. Observers judged the motion direction of stimuli in four conditions in which monocular cues, binocular cues, combined (monocular and binocular) cues, or the combined cues plus motion parallax were presented in a randomized blocked order. On each trial, they were provided feedback about their performance. Observers varied in their sensitivity to monocular and binocular cues, and showed greater sensitivity when the cues were combined. Sensitivity to both monocular and binocular cues significantly improved with feedback-based experience in the VR experiment. However, for binocular cues, the improvement had a more fundamental impact. Consistent with the hypothesis that observers without prior VR experience may discount binocular cues, the majority of observers tested early in the experiment appeared insensitive to binocular cues presented in isolation. In contrast, the majority of observers exhibited sensitivity to binocular cues when tested later in the experiment. This result is consistent with feedback-based VR experience leading the observers to stop discounting the cues. Furthermore, the addition of motion parallax appeared to cause the observers to rely almost exclusively on monocular cues. This result may be a consequence of viewing geometry, since the observers’ head movements tended to make the monocular cues available to one eye more reliable than the other cues [23]. These results help explain poor sensitivity to binocular MID cues reported in previous studies and underwhelming reactions to 3D media. In particular, providing naturally occurring visual cues does not guarantee that observers will immediately exploit those cues, even in immersive VR environments.

## Methods

### Observers

Ninety-five members of the University of Wisconsin-Madison community gave informed written consent. Stereoacuity was assessed using the Randot Stereotest (Stereo Optical Co., Inc.). All observers passed the Randot Forms test and achieved a stereoacuity of at least 100 arcsec in the Randot circles test. Five observers did not complete the experiment due to technical issues (*n =* 3), experimenter error (*n =* 1), or difficulty seeing the stimuli (*n =* 1). Data from ten observers who completed the study were excluded from analysis because they either reported prior VR experience (*n =* 4) or did not achieve above chance performance in any of the MID conditions (*n =* 6). Therefore, data from 80 observers were included for analysis. We determined that at least 52 observers would be needed to detect an effect size of .8 typical of prior results in our laboratory with 80% power at a significance level of α = .05 (two-tailed). All observers were naive to the purpose of the study. Experimental procedures were approved by the University of Wisconsin-Madison Institutional Review Board and carried out in accordance with the Declaration of Helsinki.

### Apparatus and display

**Fig 1:**
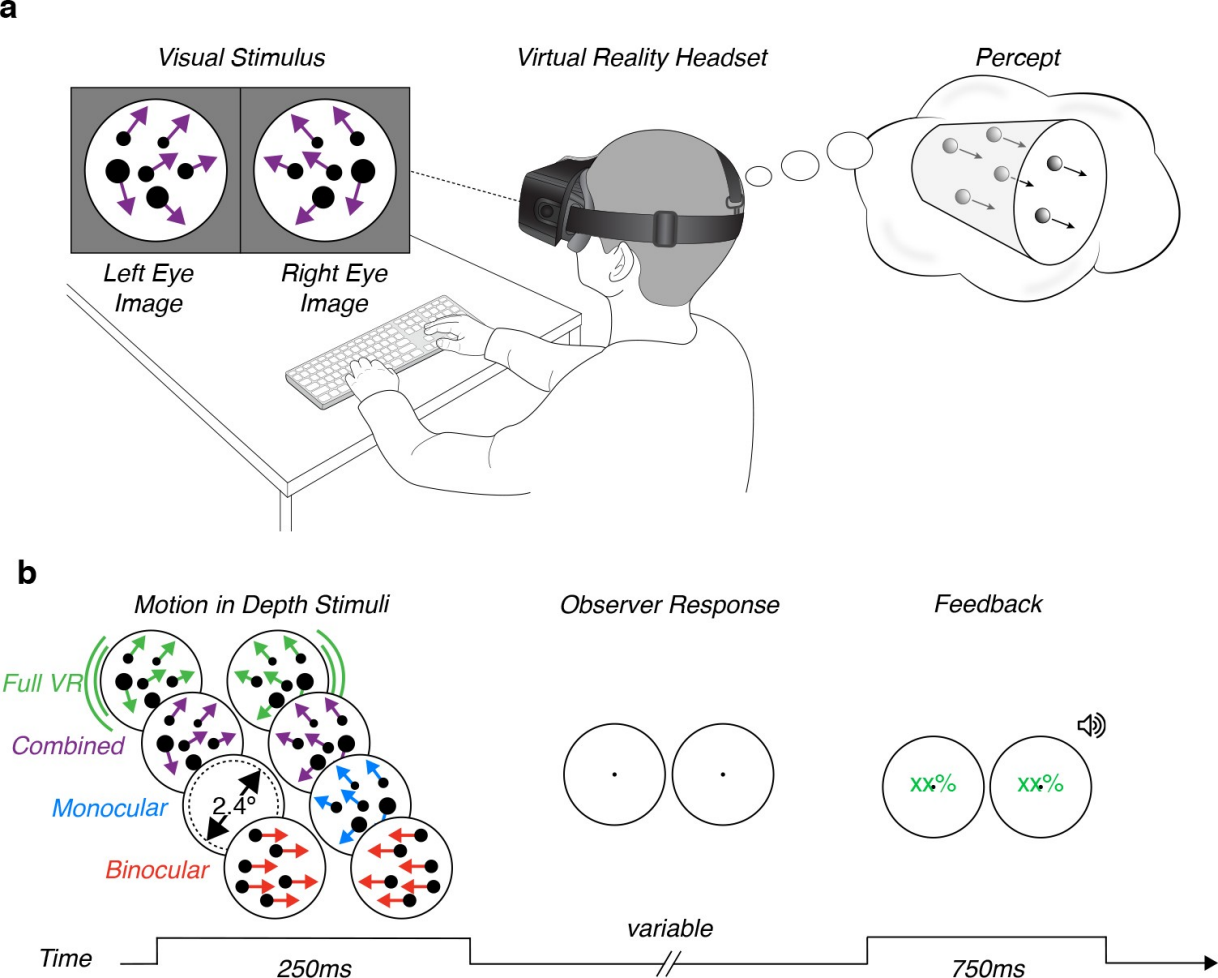
Task schematics. **(a)** Experimental setup. Observers viewed MID stimuli in a virtual reality headset. The stimuli simulated dots moving toward/away from the observer through a cylindrical volume. Observers reported the perceived motion direction. **(b)** Motion-in-depth stimuli and temporal sequence. Binocular cues stimuli contained binocularly opposite horizontal motion cues. Monocular cues stimuli contained optic flow patterns shown to one eye only. Combined cues stimuli contained both cues at the same time. Full VR stimuli contained binocular and monocular cues, as well as motion parallax. Stimuli were presented for 250 ms. Auditory and visual feedback were provided to observers after they responded. The next trial started 750 ms after feedback. The number of dots, their sizes, and optic flow patterns shown here are intended to convey the MID cues, rather than to portray the exact stimuli. Movies illustrating the actual stimuli are provided in the Supporting Information.

Observers viewed the stimuli in an Oculus Rift Development Kit 2 (DK2), a stereoscopic head-mounted VR system (Fig 1A) with a 14.5 cm low-persistence AMOLED screen (Samsung) embedded in the headset providing a resolution of 1920x1080 pixels (960x1080 pixels per eye) with a refresh rate of 75 Hz. The horizontal field of view of the device was ~85° (100° diagonal). Stimuli were presented at a simulated viewing distance of 120 cm, the focal distance of the display. Prior to the experiment, the display was calibrated for each observer’s interpupillary distance. Observers were free to move their head but were instructed to sit still. The stimuli were rendered in MATLAB (MathWorks, Inc., R2015a) using the Psychophysics Toolbox 3 [31].

### Stimuli

We presented random dot stimuli that moved either toward or away from the observer. Stimuli were presented in a 3° diameter aperture at the center of the visual field (Fig 1A). A stimulus consisted of a volume containing 12 bright (64.96 cd/m^2^) dots on a dark (0.01 cd/m^2^) background. Each dot was 0.1° in diameter when located at the fixation distance. Dots were initialized with random *x*, *y*, and *z* positions and moved either toward or away from the observer through a cylindrical volume perpendicular to the fixation plane. The volume spanned ±0.15° of horizontal disparity (a depth range of ~24 cm at the 120 cm viewing distance). Stimuli moved through the entire length of the volume during the 250 ms presentation time, producing an average retinal speed of 1.2°/s. If a dot reached a disparity of ±0.15°, it wrapped to the opposite end of the volume. Dot wrapping can cause an apparent MID signal in the direction opposite of the intended stimulus direction. To reduce this effect, each dot was assigned new *x* and *y* positions when it wrapped. To ensure binocular correspondence was not interrupted due to occlusion of a dot by the edge of the aperture, the *x* and *y* dot coordinates were restricted to lie within the central 2.4° of the aperture (Fig 1B).

To assess MID sensitivity, we manipulated motion coherence by varying the proportion of signal to noise dots. On each stimulus frame, we randomly selected a subset of dots as signal dots, which moved coherently. The remaining dots (noise dots) were assigned random *x*, *y*, and *z* coordinates within the cylindrical volume. Signal and noise dots were selected on a frame-by-frame basis to discourage observers from tracking the direction of motion of individual dots. Directionally-signed motion coherences of ±1, ±0.5, and ±0.17 were presented (corresponding to 12/12, 6/12, and 2/12 signal/total dots, respectively). The stimuli appeared as a cloud of dots with signal dots moving perpendicular to the screen and noise dots moving randomly [23].

To help with observer immersion, the stimuli were displayed in the center of a virtual room (3 m in height, 3.52 m in width, and 3.6 m in depth). The virtual walls, ceiling, and floor were mapped with tiles (except for the back wall, which was dark). The wall, ceiling, and floor elements were visible if the observer moved their head but were typically out of view during MID trials.

To facilitate version and vergence, we presented the MID stimuli within a 3° diameter circular aperture cutout of a 1/*f* noise textured panel (1.76 m x 1.76 m). The textured panel was oriented perpendicular to the stimulus motion direction and was located at the simulated viewing distance. A fixation point was shown in the center of the aperture between, but not during, trials. We instructed observers to maintain fixation on this point when it was present but did not enforce fixation.

We presented stimuli in three cue conditions in which the direction of MID was defined by monocular cues, binocular cues, or both cues combined (Fig 1B). A fourth condition included monocular and binocular cues, as well as motion parallax (made available by enabling the VR system’s capacity to update the visual display according to head position). Movies of all conditions are found in the Supporting Information.

Combined MID cues were created using projective geometry and stereoscopic presentation. Full VR MID cues were created the same way, however, head movements resulted in a corresponding change in the viewpoint of the scene, as occurs in the real world. Monocular MID cues were provided by changes in retinal dot size and the pattern of optic flow due to projective geometry. For these stimuli, binocular cues were eliminated by presenting single eye views of the combined cues stimuli [1,23]. The virtual room and 1/*f* noise texture were visible to both eyes. Binocular MID cues included interocular velocity differences (IOVD) and changing disparity (CD) signals. For these stimuli, monocular cues that signal MID were eliminated by: (1) horizontally translating the left and right eye dot pairs with equal and opposite speeds (1.2°/s), (2) using orthographic projection, and (3) drawing the dots with a fixed size (0.1° of visual angle) regardless of the simulated distance. Thus, there was no perspective information signaling MID in the binocular cues condition.

### Procedure

Each of the cue conditions was presented in a separate experimental block. The order was randomized and counterbalanced across observers. Each block contained 15 repetitions of two MID directions and three motion coherences (15×2×3 = 90 trials). On each trial, a stimulus was displayed for 250 ms. There was no time restriction on observer responses. Auditory and visual feedback were provided following each response (Fig 1B). When observers responded correctly, a “cowbell” sound was played. If they made a mistake, a “swoosh” sound was played. A running tally of the observer’s performance (percent correct) was displayed at the center of the stimulus aperture. If the observer responded correctly, the percent correct was displayed in green, otherwise in red. A new trial began 750 ms after the onset of feedback. All blocks were completed in a single session and were preceded by 5–10 trials of the full VR condition with experimenter guidance to familiarize the observer with the task and response keys. Observers reported the direction of MID (toward or away) on each trial using the up (away) and down (toward) arrow keys on a computer keyboard. The 1/*f* noise pattern of the textured panel changed between blocks to reduce perceptual fading.

### Data analysis

For each cue condition, we calculated the proportion of ‘toward’ responses as a function of the directionally signed motion coherence. We fit the data with a psychometric function (a cumulative Gaussian, *g*(*x*)), allowing for a non-zero lapse rate [23, 32] using maximum likelihood estimation in MATLAB:

$$g(x)=\lambda +(1-2\lambda )\frac{1}{2}[1+\mathrm{erf}\left(\frac{x-\mu }{\sigma \sqrt{2}}\right)],$$ Eq 1

where *x* is the directionally signed motion coherence, *μ* is the observer bias, *σ* reflects the precision of the responses, and *λ* is the lapse rate. To stabilize fits when precision was low, we enforced a bound of ±0.5 on *μ*. We assumed a maximum lapse rate of 2%. Sensitivity (1/*σ*) was our measure of interest. We enforced bounds on *σ* such that sensitivity was constrained between 0.01 and 100. To ensure stable estimates, we computed median fit parameters using a bootstrap procedure. For each observer, we resampled the “toward”/“away” response data with replacement 1,000 times and fit a psychometric function to each resampled data set. We then obtained median parameters from the fits.

To identify sensitivities associated with above chance performance, we bootstrapped a 95% confidence interval on the sensitivity of a simulated observer who responded randomly [23]. We simulated 10,000 data sets in which the responses at each coherence level had a 50% chance of being toward or away. We then fit psychometric functions to the 10,000 simulated data sets and obtained the sensitivity from each. We classified sensitivities above the upper 95% confidence bound of the simulated data (0.43) as above chance performance. Observers were included in the analyses if they performed above chance in at least one of the four conditions (*n* = 6 excluded).

In the monocular cues condition, we pseudo-randomly presented stimuli to one eye on each trial. Left and right eye sensitivities were not significantly different (Wilcoxon signed rank test: *Z* = 1.46, *p* = .14), so we merged the responses to left and right eye presentations and estimated a single measure of monocular cue sensitivity [1].

It is unknown how left and right eye monocular MID cues contribute to observer sensitivity. At one extreme is the possibility that left and right eye monocular representations are completely redundant. In that case, if monocular and binocular cue representations are independent and optimally integrated using maximum likelihood, combined cue sensitivity would be:

$$\frac{1}{\sigma_{\hat{c}}}=\sqrt{\frac{1}{\sigma_{M}^{2}}+\frac{1}{\sigma_{B}^{2}}},$$ Eq 2.1

where $\frac{1}{\sigma_{\hat{c}}}$ is the optimal combined cue sensitivity, and $\frac{1}{\sigma_{M}^{2}}$ and $\frac{1}{\sigma_{B}^{2}}$ are the monocular and binocular cue reliabilities (squared sensitivities), respectively. We refer to this as the ‘two-cue model’.

At the other extreme, left and right eye monocular cues might make independent contributions to MID sensitivity. In that case, if monocular and binocular cue representations are independent and optimally integrated using maximum likelihood, combined cue sensitivity would be:

$$\frac{1}{\sigma_{\hat{c}}}=\sqrt{\frac{1}{\sigma_{M_{L}}^{2}}+\frac{1}{\sigma_{M_{R}}^{2}}+\frac{1}{\sigma_{B}^{2}}},$$ Eq 2.2

where $\frac{1}{\sigma_{M_{L}}^{2}}$ and $\frac{1}{\sigma_{M_{R}}^{2}}$ are the left and right eye monocular cue reliabilities (which we assumed to be equivalent since the sensitivities did not significantly differ), respectively. We refer to this as the ‘three-cue model’.

The two- and three-cue models define a range of sensitivities between which left and right eye monocular signals make partially independent contributions to MID perception [5]. Upper- (defined by the three-cue model) and lower- (defined by the two-cue model) bounds of optimal combined cue sensitivity were estimated using the observer averaged sensitivities to monocular and binocular cues.

## Results

### MID cue sensitivity in observers inexperienced with VR

**Fig 2:**
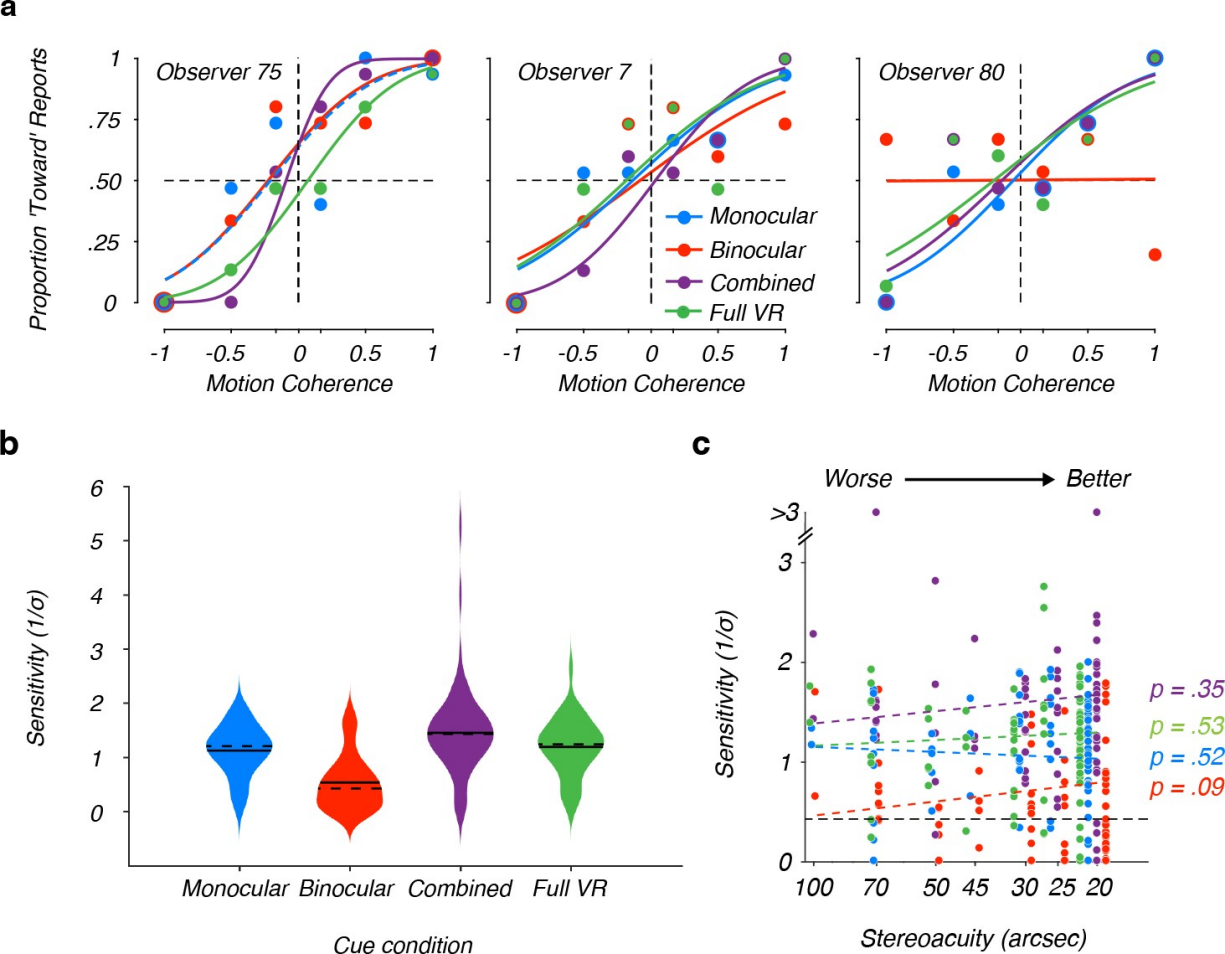
Sensitivity to MID cues. **(a)** Representative observers. Sensitivity to the visual cues that signal MID varied across individuals. Positive (negative) coherences indicate that signal dots moved toward (away from) the observer. Curves are cumulative Gaussian fits to the psychometric data. To visualize overlapping figure elements, we dashed some lines and enlarged some data points. (Left) Observer with relatively high sensitivity in all four conditions. (Center) Observer with poorer sensitivity in all four conditions. While sensitivity for these two observers differed, sensitivity in the combined cues condition was greater than when either cue was presented in isolation. This pattern indicates cue integration and was shared by the majority of observers. However, sensitivity in the full VR condition, which added motion parallax, was often less than combined cues sensitivity. (Right) Example observer sensitive to monocular cues, combined cues, and full VR conditions, but not binocular cues alone. **(b)** Comparison of monocular, binocular, combined, and full VR sensitivities across our sample (n = 80). The width of the shaded areas in the violin plots represents the proportion of the data at that level of sensitivity. The solid line in each shaded area marks the mean sensitivity and the dashed line marks the median sensitivity. **(c)** Relationship between stereoacuity and sensitivity to MID cues. Static stereoacuity did not predict sensitivity to MID based on monocular (blue), binocular (red), combined (purple), or full VR (green) cues. The horizontal black dashed line corresponds to the upper sensitivity bound associated with chance performance. Stereoacuity is plotted on a logarithmic scale, and datapoints are jittered at each stereoacuity level for visualization.

Sensitivity to each of the cue conditions varied across observers. Behavioral performance for three representative observers is shown in Fig 2A. Some observers, like Observer 75 in the left panel of Fig 2A showed relatively high sensitivity across all cue conditions, while other observers were less sensitive (e.g., Observer 7 in the center panel of Fig 2A). Although overall sensitivity differed between these two observers, both showed greater sensitivity in the combined cues condition than in either of the cue-isolated conditions, consistent with cue integration. Surprisingly, sensitivity in the full VR condition was lower than in the combined cues condition for both of these observers. As shown below, this finding was characteristic of our sample. Lastly, some observers that were sensitive to the monocular cues, combined cues, and full VR conditions seemed insensitive to MID based on binocular cues (e.g., Observer 80 in the right panel of Fig 2A). This apparent insensitivity to binocular cues was characteristic of a large portion of our sample. Indeed, across all binocular cues blocks, 50% (40/80) of observers did not perform above chance.

The pattern of sensitivities across observers is summarized in Fig 2B. Sensitivity was greatest in the combined cues condition (*M*_*combined*_ *=* 1.45; *SD*_*combined*_ *=* 0.78). An intermediate level of sensitivity was observed in the monocular cues (*M*_*monocular*_ *=* 1.13; *SD*_*monocular*_ *=* 0.47) and full VR conditions (*M*_*fullvr*_ *=* 1.19; *SD*_*fullvr*_ *=* 0.53). Sensitivity was poorest in the binocular cues condition (*M*_*binocular*_ *=* 0.54; *SD*_*binocular*_ *=* 0.50).

To test for differences between cue sensitivities, we fit a two-factor linear mixed effects model to the full dataset of measured sensitivities (n = 80 observers x 4 sensitivities = 320 data points) with cue condition and block number as fixed factors. The main effects of cue condition and block were both significant (*F*(3,313) = 62.374, *p* < .0001, and *F*(3,313) = 10.043, *p* < .0001 respectively). We first examined the main effect of cue condition. All post-hoc paired-sample t-tests revealed significant differences in observer sensitivity (*p* < .0083, the Bonferroni-corrected alpha-level), except for monocular cues vs. full VR (*p* = .24). Combined cues sensitivity was significantly greater than monocular cues and binocular cues sensitivities, consistent with cue integration.

Only 50% (40/80) of the observers performed above chance in the binocular cues condition, compared to 88.75% of observers (71/80) in the monocular cues condition. One possible explanation for poor binocular cues sensitivity is that sensitivity to binocular MID cues is limited by stereoacuity. However, given that the binocular disparity in our MID stimuli (±0.15° of disparity) greatly exceeded the stereoacuity of even our worst observers (100 arcsec, or equivalently 0.03°), it is unlikely that stereoacuity was a limiting factor [24]. Indeed, stereoacuity did not predict sensitivity to any of the conditions (all *p* > .09; Fig 2C). Thus, limitations in binocular disparity processing did not account for poor performance with binocular MID cues.

Somewhat surprising was that adding motion parallax in the full VR condition had a negative impact on sensitivity compared to the combined cues condition. In particular, sensitivity was reduced to roughly the level of the monocular cues condition. Depending on the visual field location and direction of 3D motion, the monocular cues available to one eye can be substantially more reliable than the other cues. Under such conditions, performance with combined cues can be dominated by the more reliable monocular cue [23]. We hypothesized that the decreased sensitivity was not due to motion parallax *per se*. Instead, the observers’ self-motion changed the viewing geometry, resulting in large differences in the reliabilities of the left and right eye monocular cues. We therefore quantified the effect of the head’s 3D pose on the left and right eye signal strengths, noting that the observers’ movements did not result in the occlusion of dots by the 1/*f* noise textured panel in either eye. For each trial, we computed the mean 3D head position (*x-y-z* translations) and orientation (yaw, pitch, and roll) during the 250 ms trial period. Given the head pose and interpupillary distance, we calculated the retinal speed of a dot traversing the center of the stimulus volume for each eye. On average, the retinal speeds in the two eyes differed by a factor of 1.79 (larger retinal speed = 0.84°/s; smaller retinal speed = 0.47°/s). This difference may have created a large enough difference in the reliabilities of the left and right eye monocular cues that the observers relied predominantly on the faster signal.

In sum, we found variability in sensitivity to different MID cues across a sample of inexperienced VR observers, relatively poor sensitivity to binocular cues, and evidence for monocular and binocular cue integration. Moreover, we found that an observer’s 3D pose can differentially affect left and right eye signal strengths, which can impact the relative contributions of different visual cues to MID perception. We next explored the role of feedback-based VR experience on sensitivity to MID cues.

### Experience-dependent changes in MID cue sensitivity

To assess the effect of feedback-based VR experience on sensitivity to MID cues, we examined the main effect of block number from the linear mixed effects model. We first considered the effect of exposure on sensitivity using post-hoc paired-sample t-tests to compare sensitivity across blocks independent of cue condition. Sensitivity differences between block 1 and block 3 as well as between block 1 and block 4 were significant (*p* < .001 for both, Bonferroni-corrected alpha-value = .0083). There were no other significant differences (*p* > .03 for all others). Thus, under the conditions used here, two blocks of 90 trials with feedback were sufficient to see significant improvements in MID cue sensitivity in VR.

**Fig 3:**
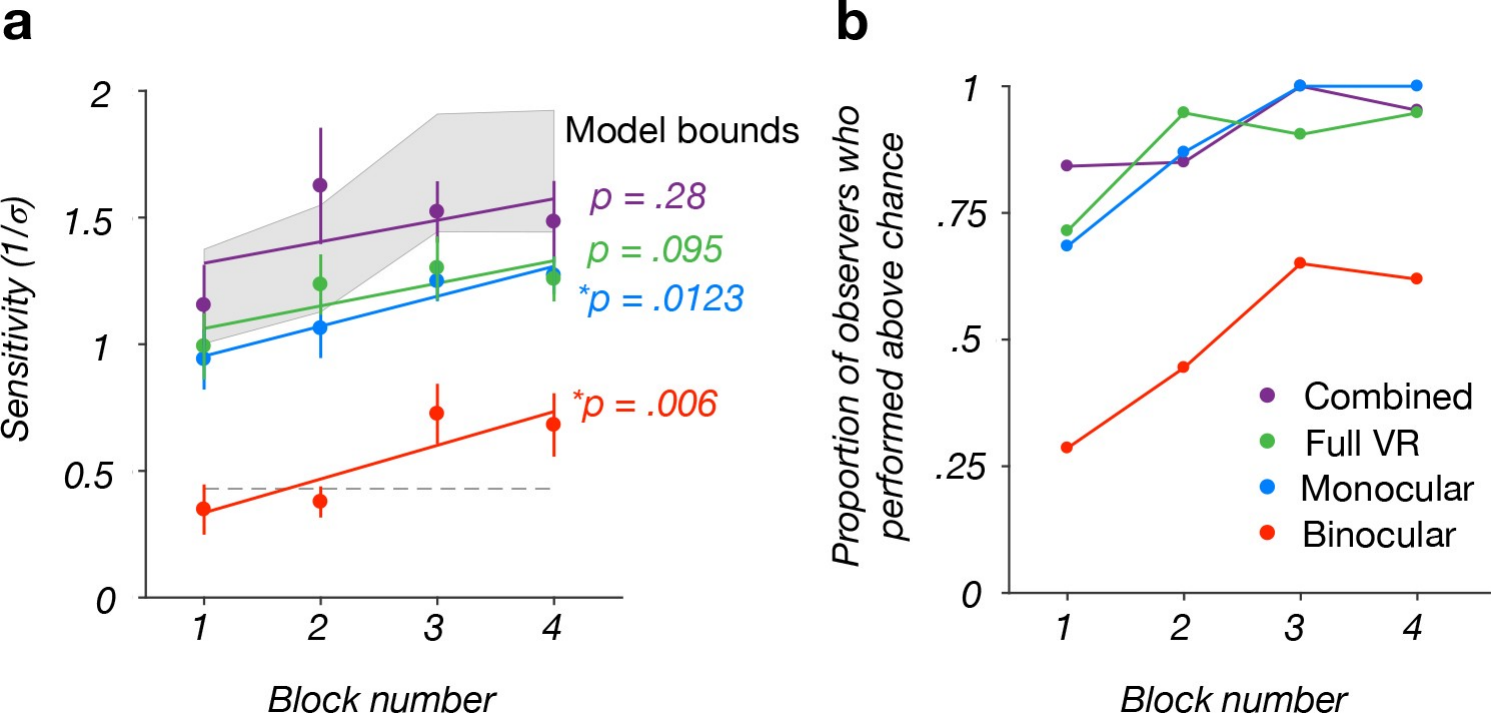
Relationship between sensitivity and experimental block number. **(a)** Sensitivity to MID based on monocular cues (blue) and binocular cues (red) significantly improved with experimental block. Numerically similar trends were observed in the combined cues (purple) and full VR (green) conditions, but the improvements were not significant. Data points correspond to between-subject mean sensitivities. Error bars correspond to ±1 standard error of the mean (SEM). Lines are regression fits. The shaded area marks the range of sensitivities between the two-cue (lower-bound) and three-cue (upper-bound) model predictions (see Results for details). **(b)** Proportion of observers who performed above chance in each condition as a function of block.

We next assessed how feedback-based experience in the VR experiment affected sensitivity in each cue condition. To address this, we carried out a linear regression for each condition to describe the relationship between sensitivity and block (Fig 3A). We found significant improvements in the binocular cues (regression line slope: β *=* .133, *t*(78) = 2.85, *p =* .006, Bonferroni-corrected alpha-value = .0125) and monocular cues (β *=* .118, *t*(78) = 2.56, *p =* .0123) conditions, but not the combined cues or full VR conditions (both *p >* .09). The difference between the average sensitivity in block 4 and block 1 for the binocular cues (Δ sensitivity = 0.334) and monocular cues (Δ sensitivity = 0.331) conditions were nearly equivalent. Similarly, the difference between the average sensitivity in block 4 and block 1 for the combined cues was 0.329. Lastly, the difference between the average sensitivity in block 4 and block 1 for the full VR condition was 0.27. Thus, sensitivity improved with feedback-based VR experience in all conditions, but the improvements were only significant in the monocular cues and binocular cues conditions.

Together, these results indicate that sensitivity to both binocular cues and monocular cues improved with feedback-based VR experience. However, when we considered the proportion of observers sensitive to each cue as a function of the block number, the impact on binocular cues sensitivity stood out (Fig 3B). For the monocular cues, combined cues, and full VR conditions, the vast majority of observers performed significantly above chance regardless of which block the condition was presented. For the binocular cues condition, however, the majority of observers performed at chance level in the first two blocks. Only in blocks 3 and 4 did a majority of observers perform above chance. These results suggest that the feedback-based VR experience may have reduced the observers’ tendency to discount binocular cues in the display.

We ruled out an alternative explanation for sensitivity differences to binocular cues across blocks. Observers who by chance happened to complete the binocular cues condition in blocks 3 and 4 might have been more sensitive to MID in general. We compared the monocular cues, combined cues, and full VR sensitivities based on the block in which the binocular cues condition was completed and found no significant differences in sensitivity between observers who completed the binocular cues condition in the first two blocks versus the last two blocks: monocular: *Z* = -1.16, *p* = .10; combined: *Z* = -.30, *p* = .76; full VR: *Z* = -1.99, *p =* .05, at the Bonferroni-corrected alpha-value = .0167.

In sum, we found that sensitivity to binocular cues and monocular cues to MID improved with feedback-based VR experience. However, that experience had fundamentally different effects on the sensitivities measured for the two cues. Only in the binocular cues condition were the majority of inexperienced observers unable to distinguish the direction of MID above chance level. This finding is consistent with the hypothesis that observers discount binocular cues when they are first encountered in a VR environment, and that discounting is reduced by feedback-based VR experience in which MID is signaled stereoscopically.

### Observers integrate monocular and binocular MID cues

We lastly tested if we could predict observers’ combined cues sensitivity based on their sensitivities to monocular cues and binocular cues. We considered two models. In the ‘two-cue model’, left and right eye monocular cues were assumed to have completely redundant representations. This model predicts that sensitivity based on both eyes’ monocular cues will be equal to the sensitivity based on a single eye’s monocular cues [1,5]. The monocular cues and binocular cues were further assumed to produce independent estimates that are optimally integrated (Eq 2.1). This model accounts for the perception of 3D surface orientation based on binocular cues and monocular cues [1–3]. Another possibility is that two independent monocular MID estimates (one from each eye) are both integrated with an independent binocular MID estimate in a ‘three-cue model’ (Eq 2.2). Partial dependence of the two eyes’ monocular cues would result in sensitivities intermediate to the two-cue and three-cue models. The two models thus define sensitivity bounds, assuming independent monocular and binocular estimates. If the monocular and binocular estimates are not independent, combined cues sensitivity would be lower.

Average observer sensitivity to the combined cues across all blocks (*M =* 1.45; *SD =* 0.78) fell within the bounds defined by the two models: [1.31, 2.01]. The observed sensitivity was not significantly different from the two-cue prediction in any individual block (all *p* > .04, with Bonferroni-corrected alpha-level = .0063; see Fig 3A). The observed sensitivity was also not significantly different from the three-cue prediction in blocks 1, 2, or 4 (all *p >* .01), but was lower in block 3 (*p* = .0048, with Bonferroni-corrected alpha-level = .0063). Together, the current findings indicate that monocular cues and binocular cues are integrated to improve the precision of MID perception, and suggest that left and right eye monocular cues may make partially independent contributions to MID estimates.

## Discussion

We investigated sensitivity to MID cues in a large cohort of observers who were inexperienced with VR. Consistent with previous results, we found that sensitivity to the visual cues that signal MID varied across individuals [23]. Sensitivity was greater when monocular cues and binocular cues were presented together than in isolation, indicating cue integration.

While the majority of observers performed well above chance in the monocular cues, combined cues, and full VR conditions, half of the observers appeared insensitive to binocular cues. Previous work similarly reported relatively poor sensitivity to binocular MID cues, especially in inexperienced observers [23,27–28]. Poor sensitivity to binocular cues may be due in part by observers’ prior experience with 2D displays which convey MID using monocular cues only. Indeed, stimuli that only depict MID through binocular cues provide conflicting information since they will also contain monocular cues that signal that the stimuli are constrained to the image plane at all times (e.g., no size changes). Such conflicts do not appear to affect sensitivity to binocular cues for static 3D orientation judgments [1], but may be more prevalent with moving stimuli. Thus, poor sensitivity to binocular cues may partially reflect the discounting of binocular MID cues in 3D displays.

We hypothesized that feedback-based VR experience would lead observers to stop discounting binocular cues. Experience in the VR task was associated with significant improvements in sensitivity to both monocular cues and binocular cues. However, there was a fundamental difference in how sensitivities to these cues were affected. With monocular cues, the majority of observers showed significant sensitivity in all four blocks. In contrast, the majority of observers performed at chance level in the binocular cues condition in blocks 1 and 2. Only in blocks 3 and 4, did a majority show significant binocular cues sensitivity. Sensitivity to binocular cues remained substantially weaker than sensitivity to monocular cues throughout the experiment. We note, however, that sensitivity to binocular MID cues is speed dependent [33,34], so the relative sensitivity to monocular and binocular cues may also be speed dependent.

We found that monocular and binocular cues are integrated to improve the precision of MID perception. In three out of the four experimental blocks, the average observer sensitivities were in-between the predictions of a two-cue model (in which a single monocular cues representation was integrated with an independent binocular cues representation) and a three-cue model (in which left eye monocular, right eye monocular, and binocular cues make independent contributions to perception). This result is consistent with the possibility that left and right eye monocular signals make partially independent contributions to MID perception. This contrasts with static 3D orientation perception, which is consistent with the two-cue model [1–3]. If subsequent data support this difference, it would suggest that left and right eye monocular signals may be integrated differently within the neural circuits supporting 3D orientation and motion processing.

Since the current study was performed with inexperienced observers and few motion coherence values, sensitivity estimates were sometimes noisy. Furthermore, as is a common limitation with cue-combination studies, discrepancies between observed and predicted sensitivities may arise in part due to underestimating sensitivity to cue-isolating conditions or ceiling effects in estimates of sensitivity to combined cues. Future work that obtains more precise estimates from experienced observers can help further elucidate how different MID cues are perceptually integrated.

We previously found that the performance of inexperienced observers in a MID task only improved with feedback [30]. However, we could not identify whether the improvements were due to changes in sensitivity to monocular and/or binocular cues because the cues were not presented in isolation. The current results suggest that feedback likely improves sensitivity to both cues.

Enabling motion parallax cues had a surprisingly negative effect on sensitivity. Our analysis revealed that changes in observer head position resulted in retinal speeds in the two eyes that differed by a factor of 1.79. We previously found that differences in left and right retinal speeds can result in a “winner-take-all” scenario in which combined cues perception is dominated by the single most reliable monocular cue [23]. We speculate that this effect underlies the detrimental impact of enabling motion parallax in the current study. In contrast, previous work found that head movements can enhance MID perception due to motion parallax above and beyond perception due to the combination of monocular and binocular cues [30]. However, there are a number of differences between these studies. In the previous study, a single target moved for 1000 ms as opposed to multiple dots for 250 ms, reducing the availability of monocular optic flow (expansion/contraction) cues, and increasing the availability of motion parallax cues. The stimuli in the previous study also moved along a variety of trajectories in the *x-z* plane and contained much larger disparities (up to ~1.2°), compared to the current study in which only toward and away motions were shown with a disparity range of 0.3°. Future work can evaluate how factors such as the stimulus configuration and presentation time affect the relative contributions of MID cues, and in particular the effect of motion parallax on MID perception.

In conclusion, these findings suggest a reinterpretation of the performance of inexperienced observers. Rather than interpreting poor sensitivity to binocular MID cues as a deficiency in stereoscopic processing, poor sensitivity may reflect a reasonable discounting of binocular cues given their experience with visual displays. We speculate that feedback-based experience with stereoscopic displays may update observers’ expectations about the 3D information that can be contained in visual displays. Similar effects may occur with other cues that are typically absent in visual displays but can be provided in virtual and augmented reality environments, such as motion parallax and accommodative blur. Until prior expectations are appropriately updated, observers may be limited in their ability to take advantage of those cues, even if they rely on them in the natural environment.

## Supporting information

S1 Movie Movie illustrating the monocular cues stimulus condition. (MP4)

S2 Movie Movie illustrating the binocular cues stimulus condition. (MP4)

S3 Movie Movie illustrating the combined cues stimulus condition. (MP4)

S4 Movie Movie illustrating the full VR stimulus condition. (MP4)
